# Prevalence of Parental Comments on Weight/Shape/Eating amongst Sons and Daughters in an Adolescent Sample

**DOI:** 10.3390/nu13010158

**Published:** 2021-01-06

**Authors:** Lucy Dahill, Deborah Mitchison, Natalie M. V. Morrison, Stephen Touyz, Kay Bussey, Nora Trompeter, Alexandra Lonergan, Phillipa Hay

**Affiliations:** 1 Translational Health Research Institute, School of Medicine, Western Sydney University, Sydney, NSW 2715, Australia; deborah.mitchison@westernsydney.edu.au (D.M.); n.morrison@westernsydney.edu.au (N.M.V.M.); p.hay@westernsydney.edu.au (P.H.); 2 Centre for Emotional Health, Department of Psychology, Macquarie University, Sydney, NSW 2109, Australia; kay.bussey@mq.edu.au (K.B.); nora.trompeter@mq.edu.au (N.T.); alexandra.lonergan@hdr.mq.edu.au (A.L.); 3 InsideOut Institute, The University of Sydney, Sydney, NSW 2006, Australia; stephen.touyz@sydney.edu.au; 4 Campbell and Campbelltown Hospitals, South West Sydney Local Health Districts, Campbelltown, NSW 2560, Australia

**Keywords:** mother, father, weight talk, prevalence, adolescents, parent–adolescent communication

## Abstract

Reports suggest that 12–76% of adolescents have experienced parental comments regarding their weight/shape and/or eating behaviours. Parents may engage in conversations about weight/shape and eating out of concern, even without any ill intent; however, the associations of these comments with subsequent problematic psychosocial and eating behaviours are evidenced. Therefore, an in-depth understanding of the content and prevalence of such comments is needed. To date, adolescent-reported prevalence estimates have not included differentiation between mother or father and sons or daughters, nor have they considered eating-focussed comments. This study considered the prevalence of positive and negative parental commentary regarding weight/shape and eating with a focus on parental origin. A total of 2287 Australian male and female adolescents participated via a self-report survey. Adolescents reported frequent positive comments on weight/shape and on eating, most commonly maternal positive comments on weight/shape (78%; 95% CI 77–80). Daughters reported significantly more maternal comments on weight/shape (positive and negative) as well as more negative eating comments from mothers than did sons. Sons reported significantly more negative weight/shape comments from fathers than did daughters. Some negative comments increased significantly with age. These findings support a notable prevalence of reported parental weight/shape and eating comments directed at their offspring, particularly from mothers.

## 1. Introduction

Parent weight-based talk, which includes “fat-talk” (negative communication about weight), comments and teasing about appearance, weight and shape, conversations about dieting and healthful eating suggestions, is common during adolescence [1,2,3]. It is known that parental comments about weight, shape and eating can have negative associations with adolescents such as weight gain, binge eating and unhealthy weight control behaviours and mental health such as shame, depression and weight bias internalisation [4,5,6]. Nonetheless, considering the high prevalence of different types of weight-related problems among adolescents to date [7], little attention has been directed to general population estimates of prevalence or to understanding the full range and complexities of comments, including their negative/positive valence, parental origin (i.e., from the adolescent’s mother or father), the gender of the adolescent themselves (i.e., a daughter or a son) and the content of the comments (i.e., about weight, shape, appearance or eating) and demographic features such as gender and age. Understanding these complexities will assist in the development of interventions targeting these potential risk factors.

### 1.1. Adolescence

Adolescence, defined by the World Health Organization as between 12 and 19 years of age [8] is a key period for social, emotional and cognitive development when behavioural patterns are set that may continue into later life [2,3,9,10]. This foundational stage in life has varied and dynamic physical, social and emotional changes, and the role and influence of parents often changes considerably in this time, with adolescents striving for independence as they move through early (11–13 years of age), middle (14 to 16 years of age) and later (17 to 19 years of age) stages [10]. Parent–child connectedness is an important protective factor for common mental health problems, such as depressive symptoms [11], which can be challenged by changes in family structure such as single and blended family structures during this foundational stage, and different parenting styles and “incendiary” parent–adolescent conversation which Parletta et al. suggest may contribute to disordered eating [12]. Individual studies and a meta-analysis that have considered the prevalence of critical or other comments and associations with eating disorders and disordered eating indicate age as an important moderator of this relationship, with the relationship being stronger with increasing adolescent age [13,14,15,16,17]. Therefore, exploring age by adolescent grouping of early, middle and later adolescence as captured by school years 7–8 (early), 9–10 (middle) and 11 and 12 (later) was considered of interest in this study.

### 1.2. Prevalence

The prevalence of adolescent self-reported parental comments on weight/shape/eating is broad, ranging from 12% to 76% [3,5,13,15,18,19,20,21,22,23]. Seven relevant adolescent-report prevalence papers exist on this phenomenon to date. Of these, three emanate from the data set of Project EAT 2002 [13,19,20,21], a US-based population study of eating patterns and weight concerns amongst teenagers [20]. This study reported the prevalence of parental weight teasing, reported by boys and girls as 17% and 29%, respectively, but it did not report the breakdown of comments coming from mothers versus fathers or consider different types of comments beyond appearance (e.g., on eating). There is evidence to suggest that mixed-sex interactions between parents and their children (e.g., paternal comments to daughters) have important associations with body image and mental health outcomes [3], yet little is known about the prevalence of these interactions. Therefore, a greater understanding of the source and type of comments or teasing is relevant and will provide information regarding key targets for future interventions. Two studies have considered comments from mothers or fathers [3,15]. Keery et al.’s (2005) study assessed frequency and type (both positive and negative) of reported weight and appearance comments (both positive and negative) emanating from mothers and fathers in a sample of 372 adolescent girls. Findings indicated more overall appearance (19% vs. 13%) and weight-based (10% vs. 6%) comments from fathers than mothers. This study was limited by not including boys and did not consider comments on eating. A study by Almenara and Jezek (2015) also considered the breakdown between parental genders (mother 19% vs. father 13%) in a sample of 570 adolescents. Although it did not break down the type of comments, the trajectory of greater adolescent-reported maternal comments was in line with other mixed-gender population studies. Specifically, girls reported more weight and appearance comments (either positive or negative) from their parents than did boys [13,15,20,21,22,24]. Rodgers, Faure and Chabrol’s 2009 French study found girls reported more weight shape comments of either valence from their mother and more negative weight shape comments from their father [18]. Studies on prevalence have a mix of clinical and non-clinical populations [15,21,22,23]. One study had an athletic population [25] and another military-dependent population [26]. Their diversity is both a strength and a limitation, and a reduced number only included female adolescents [3,27,28]. Furthermore, there is a lack of consistency in the type of comments in these studies (i.e., those with negative compared to positive valence), few that considered parental origin of such comments (i.e., from adolescent’s mother or father) and also considered the content of the comments (i.e., about weight, shape, appearance or eating).

### 1.3. Parental Communication

During adolescence, parent–child communication can change and present challenges based not only on a changing power dynamic, which can influence how communication is received and perceived [10,29], but also on parenting styles [12] and gendered socialisation, where the gendered dyads in families can also influence outcomes and parent connectedness, which includes communication with parents [19]. Throughout the research, it is clear it is more complicated “than any one person or even any one rule or pattern” [30,31], and therefore, it is important to understand prevalence and type of parental comments (and later health associations with these comments) *without* apportioning blame to parents as parent communication is part of a web of influencing factors [32]. Berge et al. (2014) recognised that it may be natural for a parent who is concerned about their adolescent’s weight to engage in conversations about weight, shape and eating, and these conversations are most common amongst same-sex parent/child dyads [5]. However, what the parent may perceive as a weight concern is also influenced by sociocultural pressures for thin or muscular ideals, and therefore, a parent may engage in a conversation with an adolescent about their weight, shape and eating that may detrimentally add undue pressure to the child and internalisation of unattainable ideals despite the parent’s intentions [33,34,35,36]. Further, the need for parental concern and communication with their child about their child’s weight or shape may be misplaced and driven by societal messages, such as the “obesity epidemic” and parent-internalised weight bias [36]. Longitudinal evidence of patterns for communication within families emerged through issue 1 of the WHO’s large international cross-sectional survey Health Behaviour of School-Aged Children (HBSC) [37]. This study found very few children reported difficulties talking to their mother, and therefore, mothers tended to play a larger role with helping children with their problems. However, communication challenges increased with age (15% amongst 11-year-olds, to 23% for 13-year-olds and 28% with 15-year-olds) in all countries. Communication with their father was more difficult and also increased with age and was consistent across all countries (33% amongst 11-year-olds, to 45% for 13-year-olds and 52% amongst 15-year-olds). This was consistent with later research by Al Sabbah et al. (2000) [38].

Perception of comments is always hard to gauge and can be influenced by a complex sphere of influence [32], self-perception and health motivation. For example, a teen exhibiting low health motivation and unhealthy eating habits may perceive they receive more challenging messages and therefore report more negative comments [39]. Klein et al. (2017) reported that paternal comments for their all-female cohort (m = 19.87, +/− 1.64), significantly predicted drive for thinness in women 20 years later [40]. Ackard et al. (2006) also found that 52.1% of girls (48.6% boys) would talk to their mother “quite a bit” or “very much” more than their father and “the majority reported they could not talk to their father (quite a bit/very much: 24.6% girls, 38% boys)”. However, the same paper highlighted that the majority of participants reported valuing their parent’s opinion for “big” decisions and experienced positive parental care. This last point was an important protector of behavioural and emotional health and was associated with fewer unhealthy weight control behaviours [19]. 

There is less research that considers both positive and negative comments on weight, shape and eating as perceived by both sons and daughters between 12 and 19 years of age from both mothers and fathers. Information on the prevalence of these communications will improve understanding of how adolescents perceive their parents’ comments on their bodies and eating habits as they move through this developmental stage.

### 1.4. Parental Comments and Age

Adolescence encapsulates a wide age range with very different physical and social–emotional developmental challenges [41]. This age also refers to a time where relationships with parents can become more conflictual and less warm, and this can influence or mediate other environmental biological or psychodynamic factors [42]. In a review of early adolescence literature on parental modelling of appearance and weight ideals, parents were found to have considerable influence over early adolescents’ appearance ideals [35,43]. This was further supported by a 2018 study which found and supports research that parental teasing increased symptoms over time [44]. There is less consistency in the research for associations in older adolescents, which may reflect that this age range (14–19 years) is when adolescents tend to transition from parents being a primary source of influence to peers playing a more significant role [42,45]. Parents can influence body dissatisfaction and disordered eating, but this influence may differ between males and females and is often mediated by internalisation of appearance ideals and appearance comparisons, which may reflect broader sociocultural and peer influences [46,47]. Thus, further research is needed to understand the change in prevalence of self-reported comments from parents on weight, shape and eating among older adolescents.

#### Aim

Thus, the current study was designed to investigate the prevalence of gendered parental comments from a large representative sample and the prevalence of positive and negative comments on weight, shape, and eating as experienced by sons and daughters and emanating from fathers versus mothers. Based on prevalence percentages in current literature and previous research, prevalence of overall comments received from parents was hypothesised to be at least 20% and greater from mothers than fathers. Due to paucity of research specific to different types of comments, content of comments and other research questions, namely gender and age differences in perceptions of comments, were exploratory.

## 2. Materials and Methods

### 2.1. Sample Characteristic

Participants of this study were a subset of Wave 2 of the EveryBODY study (*n* = 3242), a large longitudinal study on eating disorders and body image concerns in Australian adolescents. Participants were from eight schools, four private and four public in New South Wales, Australia (54% girls). Parents and students were informed of the purpose of the study, and students who participated were offered a chance to win one of 10 $100 gift cards. More detail of the recruitment and sampling is detailed elsewhere [48]. The sub-sample used in this study (*n* = 2287) includes those participants who indicated they had a mother or father in their life, completed the questions relating to parental comments (which were located towards the end of the survey) and were aged between 11 and 18 years. The mean age was 14.84 years (SD = 1.50). 

Of the 2287 adolescents asked to complete the survey, 46% reported being male and less than 1% did not report a gender or reported non-binary gender. In total, 2252 (98%) of the participants who reported a mother being in their life reported any comments from their mothers on their weight/shape and/or eating, and *n* = 2217 (93%) who reported a father being in their life reported any comments from their fathers on their weight/shape and/or eating. Participants were in grades 7 (20%), 8 (21%), 9 (22%), 10 (20%), 11 (7%) and 12 (9%). Most participants self-identified as being born in Australia (83%). Mean BMI (body mass index) percentile was 51.26 (SD = 30.33).

### 2.2. Prevalence Measure

Questions were developed for the purposes of this study to establish the prevalence of positive and negative comments from mothers and fathers to sons and daughters. There was no timeline associated with when the adolescent was recalling the comments. Participants were firstly asked whether their mother or father were in their lives, and the response type for these two questions was dichotomous (yes or no). Participants were asked “Is your mother in your life?”, described as having “regular contact with your mother”, and if they had 2 mothers in their life, they were asked to answer for the one they spent most time with. The same questions and clarification were asked related to having “regular contact with your father”. Participants who selected “yes” were asked 4 further questions relating to their mother, and then another 4 relating to their father, to examine the perceived frequency of positive and negative maternal and paternal comments, on (1) weight and shape and (2) eating. Questions were worded as follows: “How often does your mother (father) comment positively (negatively) on your weight or shape (eating)?” To measure the frequency of comments, participants responded on a 5-point Likert type-scale with response options “never” (1), “rarely” (2), “sometimes” (3), “often” (4) and “all of the time” (5). The study questions evaluated “weight/shape” as one item and “eating” separately. 

### 2.3. Procedure

Data were collected via computer-administered questionnaires during class time, supervised by their teacher. The study was approved by the Macquarie University Human Research Ethics Committee (HREC 5201600312) and the New South Wales Department of Education. 

### 2.4. Statistics 

Data were cleaned and inspected for normality. All analyses were performed using SPSS version 26. Prevalence proportions were calculated with 95% CI and inspected for overlap. A series of chi-squared tests were performed to examine the overall differences and differences across adolescent gender, year group and maternal/paternal source on reported weight/shape and eating comments. As we were particularly interested in parental comments to sons versus daughters, adolescent gender was considered a binary response and all non-binary responses (less than 1%) were excluded from the final analysis of gendered comments but included in the “all” comments from parents. Parental comments were considered present if reported as “rarely”, “sometimes”, “often” or “always” occurring. Year groups were based on the participants’ current school year, starting with Year 7 (first year of High School, students aged usually around 12 years) through to Year 12 (final year of High School, students aged around 18 years). As in our [7] and other previous research [10], we used grades 7 and 8 to indicate early adolescence, grades 9 and 10 to indicate mid-adolescence and grades 11 and 12 to indicate late adolescence. Using such year groups was based on the rationale that year cohorts develop together in important ways, e.g., socially, academically, and thus, two students of the same age will be less comparable if they are in two different year groups than two students within the same year group (even if their age slightly differs). Because of multiple testing across year groups, the alpha level was set to <0.01.

## 3. Results

### 3.1. Prevalence of All Positive and Negative Parent Comments on Weight/Shape and Eating

Table 1 displays the prevalence of parental comments across parent gender. The prevalence of positively (78%) and negatively (37%) perceived maternal comments on weight/shape was higher than the prevalence of positively (51%) and negatively (28%) perceived paternal comments on weight/shape, respectively. Likewise, positively (70%) and negatively (61%) perceived maternal comments on eating were also more commonly reported than positively (53%) and negatively (44%) perceived paternal comments on eating. 

### 3.2. Prevalence of Positive and Negative Parent Comments on Weight/Shape and Eating and Adolescent Gender

Table 2 further compares the prevalence rates of these parental comments according to the adolescent’s gender. As shown in Table 2, daughters (compared to sons) were significantly more likely to report positive (85% vs. 71%) and negative (40% vs. 33%) commentary from their mothers about their weight/shape. Conversely, sons (compared to daughters) were significantly more likely to report negative (but not positive) commentary from their fathers about their weight/shape (32% vs. 25%). Sons reported significantly fewer negative comments around eating from their mothers compared to daughters (57% versus 66%). There were no other significant differences.

As shown in Table 3 and Table 4, the frequency of perceived positive comments generally decreased with increasing adolescent stage, and the frequency of negative comments increased. However, only perceived paternal positive weight/shape and positive eating comments and maternal negative weight/shape comments reached significance. Perceived increases in negative maternal and paternal weight/shape and maternal eating comments with increased adolescent years reached significance for girls. Positive paternal eating comments reduced in prevalence with increased years for girls.

## 4. Discussion

The current study was designed to investigate the prevalence of positively and negatively perceived comments on weight, shape and eating from fathers and mothers as reported by sons and daughters during an important life stage of changing cognitive social and emotional experiences [9,10,11]. This was cognisant that such communications with parents are complex and often mediated by the quality of the relationship between family members, allowing the expression of both negative and positive comments and open communication of concerns [49]. 

### 4.1. Prevalence

Adolescents in our study reported a high prevalence of comments from parents, both positively and negatively perceived. We had estimated a prevalence of 20%, but our findings of 71% for negative eating comments and 60% for positive eating comments are more in keeping with Almenara and Jezek, who reported a prevalence of 76% in their mixed-gender study reporting appearance teasing [15]. In line with previous research, the source of the highest prevalence of comments overall (both positive and negative) was from mothers, especially in regard to their daughters [13,15,18,19,20,21,22,23]. In our findings, daughters reported more negative eating comments than sons from mothers, which could be explained by girls engaging in more appearance conversations with their mothers than boys and therefore increasing the likelihood of receiving comments of a positive or negative valence [15,19,50]. Furthermore, the data included in Menzel et al.’s meta-analysis suggested that not only do girls experience more weight-related teasing, but they are also more bothered by it, which may in turn increase its salience and likelihood of self-report [17,20]. In our study, sons were more likely than daughters to report negative comments about weight and shape from fathers, which could lend support to research that illustrates boys have more conversations in athletic domains such as muscle building [15,50]. This is an area that warrants further investigation as there is a paucity of research specifically considering the prevalence of perceived comments on body image from mothers and fathers to sons. To our knowledge, this is the first study to find sons may perceive more negative comments on eating from their fathers than do daughters.

### 4.2. Prevalence of Comments and Gender

The structure of families has changed considerably in recent years with shifts from an increase in single-parent and blended family structures [51], and our findings are in accord with previous research investigating gendered dyads within family structures and the potential for gendered influence [5,33,34,36]. Indeed, family processes suggest that men are socialised differently to women and therefore have different focuses for conversations [30] and that mother–child and father–child connectedness contributes to the seeking and valuing of opinions, and perception of comments received [19]. Al Sabbah et al.’s large 2009 international study illustrated the perception that difficulty talking to fathers was more common than difficulty talking to mothers [37,38]. This could explain the higher prevalence of maternal comments found in our study as women tend to have more conversations that build connectedness, and men are more likely to converse about finding solutions [30]. Thus, women may have more conversations based on their own influences and internalisation that can influence an adolescent girl’s biological changes, such as the increase in fat stores and menarche [42,44]. Mother and daughter comments may be influenced by their own internalised weight concerns, which are more common than in men [36,47]. This may account for the higher frequency of maternal comments. However, the high frequency of paternal comments indicates high levels of concerns and that gender alone is an incomplete explanation for the findings. Parenting styles may also be an important consideration for how communication is perceived. Where there is a more conflictual parenting style, parental comments may impact more on diet and weight status, perception of communication and this may influence gender reports [12,38,39]. 

### 4.3. Prevalence of Comments and Year Group

Our findings showed important age differences, whereby negative comments were more common and positive comments were less common in the older age groups compared to younger age groups. This is consistent with previous research by Zimmer-Gembeck et al. and Al Sabbah et al. [38,44]; however, only perceived paternal positive weight/shape and positive eating comments and maternal negative weight/shape comments reached significance. There were more significant findings for girls alone. Further research is needed to replicate these findings and also explore the questions of whether the perception of positive/negative comments decreases as adolescents age, which could explain the lower number of comments reported, whether the importance of perceived parental comments decreases with age, for example, whether they “tune out” parents’ comments, or whether there are changes in parental intentions and influence on the adolescent as they age, for example, whether adolescents get used to parenting and communication style. 

### 4.4. Strengths and Limitation and Further Research

Strengths of this study include the use of a mixed-gender, general population sample, with consideration of the gendered origin of parental comments and a breakdown of weight and shape (appearance) comments as well as specific comments on eating and the breadth of early, mid- and late adolescence considered in context of their peer influence through considering them as a school stage grouping (early—years 7 and 8, mid—years 9 and 10 and late—years 11 and 12). Limitations include the reliance on self-report and cross-sectional data (thus giving rise to recall bias) and consideration of binary gender only. Thus, findings are not generalisable to adolescents of greater gender diversity. It is also possible that low numbers may explain some of the findings for adolescent stage and perceived increases in negative comments not reaching significance (Type 2 error). Weight/shape was also the only aspect of appearance, and eating the only aspect of diet considered. Future studies should examine broader aspects of body image related comments and diet. Future studies should also seek to examine the intention of the parent when making such comments. 

Future research will need to examine whether positive comments, or the way conversations related to eating, weight and shape are communicated, are a protective or risk factor for later development of eating and/or body image problems. It is also important to recognise that parents, like adolescents, may decode their communication in a way that was not intended. Without asking the parents of the participants included in this study, we will not know if their comments were intended as positive or negative. However, the results offer valuable insight to how maternal and paternal comments are perceived by adolescents within the context that, without an understanding of how words are intended in communication and parent–adolescent connectedness, even a low level of parent teasing can be problematic [12,18,19,32,39,40,49].

## 5. Conclusions

Overall, perceived positive weight/shape and eating comments were more common than negative such comments by either parent, but negative comments were still commonly reported. Daughters perceived more positive weight/shape, as well as more negative weight/shape and eating comments from mothers than did sons, whereas sons perceived significantly more negative weight/shape comments from fathers than did daughters. It is now imperative that longitudinal research examine the potential prospective protective versus deleterious outcomes of such commentary from fathers vs. mothers and in boys and girls separately. This should include examination of which parameters in the current study (i.e., parent gender, adolescent gender, comments on eating vs. weight/shape, positive vs. negative comments) are risk or protective factors for the development of later body image and eating problems. Findings need to be replicated, and future research should ask parents about their intention with their comments, in order to explore a putative mismatch between intention and perception.

## Figures and Tables

**Table 1:** Prevalence of perceived positive and negative parent comments on weight and shape and eating (*n* = 2287 adolescents).

Perceived Parental Comment	*N*	% (95% CI)
Weight/Shape—positive		
Maternal	1790	78% (77–80)
Paternal	1169	51% (49–53)
Weight/Shape—negative		
Maternal	847	37% (35–39)
Paternal	638	28% (26–30)
Eating—positive		
Maternal	1606	70% (68–72)
Paternal	1217	53% (51–55)
Eating—negative		
Maternal	1404	61% (59–63)
Paternal	997	44% (42–46)

**Table 2:** Prevalence of any perceived positive and negative weight, shape and eating comments from 2287 daughters and sons.

Comment Frequency *	Daughters	Sons	Statistics
	*N* (%)		Chi-Square (*df*), *p*
Maternal Positive Weight/Shape	1022 (85%)	768 (71%)	85.83 (4), *p* < 0.001
Maternal Negative Weight/Shape	486 (40%)	361 (33%)	13.71 (4), *p* < 0.008
Paternal Positive Weight/Shape	621 (51%)	548 (51%)	4.54 (4), *p* < 0.338
Paternal Negative Weight/Shape	295 (25%)	343 (32%)	21.39 (4), *p* < 0.001
Maternal Positive Eating	883 (73%)	723 (67%)	11.88 (4), *p* < 0.018
Maternal Negative Eating	791 (66%)	613 (57%)	17.53 (4), *p* < 0.002
Paternal Positive Eating	647 (54%)	570 (53%)	2.21 (4), *p* < 0.699
Paternal Negative Eating	533 (44%)	464 (43%)	10.85 (4) *p* < 0.028

* Any comment frequency included Rarely, Sometimes, Often or Always.

**Table 3:** Prevalence of any perceived positive and negative maternal comments on weight/shape and eating based on adolescent stage (*n* = 2287 adolescents).

	Early (Year 7–8)	Middle (Year 9–10)	Late (Year 11–12)	*χ^2^* (*df* = 2) *p*	Bonferroni Post-Hoc *p* < 0.05
	Any comments *n* (%)				
Maternal					
Positive Weight/Shape					
All	756 (80.8%)	762 (78.5%)	307 (80.8%)	1.83, 0.400	n.s.
Girls	436 (86.3%)	420 (83.8%)	179 (89.9%)	4.54, 0.103	n.s.
Boys	320 (74.2%)	342 (72.8%)	128 (70.7%)	0.83, 0.660	n.s.
Negative Weight/Shape					
All	313 (33.4%)	378 (38.9%)	191 (50.3%)	32.38, <0.001	Early < Mid < Late
Girls	173 (34.3%)	206 (41.1%)	120 (60.3%)	39.94, <0.001	Early, Mid < Late
Boys	140 (32.5%)	172 (36.6%)	71 (39.2%)	3.06, 0.217	n.s.
Positive Eating					
All	690 (73.7%)	685 (70.5%)	266 (70.0%)	3.06, 0.217	n.s.
Girls	384 (76.0%)	365 (72.9%)	147 (73.9%)	1.37, 0.505	n.s.
Boys	306 (71.0%)	320 (68.1%)	119 (65.7%)	1.87, 0.393	n.s.
Negative Eating					
All	573 (61.2%)	607 (62.5%)	259 (68.2%)	5.70, 0.058	n.s.
Girls	319 (63.2%)	335 (66.9%)	150 (75.4%)	9.59, 0.008	Early < Late
Boys	254 (58.9%)	272 (57.9 %)	109 (60.2%)	0.32, 0.854	n.s.

n.s = not significant.

**Table 4:** Prevalence of any perceived positive and negative paternal comments on weight/shape and eating based on adolescent stage (*n* = 2287 adolescents).

	Early (Year 7–8)	Middle (Year 9–10)	Late (Year 11–12)	*χ*^2^ (*df* = 2) *p*	Bonferroni Post-Hoc *p* < 0.05
	Any comments *n* (%)				
Paternal					
Positive Weight/Shape					
All	575 (61.4%)	563 (58.0%)	201 (52.9%)	8.34, 0.015	Early > Late
Girls	318 (63.0%)	292 (58.3%)	109 (54.8%)	4.67, 0.097	n.s.
Boys	257 (59.6%)	271 (57.7%)	92 (50.8%)	4.08, 0.130	n.s.
Negative Weight/Shape					
All	308 (32.9%)	349 (35.9%)	151 (39.7%)	5.80, 0.055	n.s.
Girls	148 (29.3%)	161 (32.1%)	84 (42.2%)	10.91, 0.004	Early, Mid < Late
Boys	160 (37.1%)	188 (40.0%)	67 (37.0%)	0.95, 0.621	n.s.
Positive Eating					
All	609 (65.1%)	577 (59.4%)	201 (52.9%)	17.83, <0.001	Early > Mid, Late
Girls	336 (66.5%)	301 (60.1%)	108 (54.3%)	10.20, 0.006	Early > Late
Boys	273 (63.3%)	276 (58.7%)	93 (51.4%)	7.69, 0.021	n.s.
Negative Eating					
All	474 (50.6%)	500 (51.5%)	193 (50.8%)	0.15, 0.928	n.s.
Girls	255 (50.5%)	265 (52.9%)	111 (55.8%)	1.69, 0.429	n.s.
Boys	219 (50.8%)	235 (50.0%)	82 (45.3%)	1.62, 0.445	n.s.

n.s = not significant.

## Data Availability

Deidentified data are available for collaborative projects from the author D.M. upon request, subject to approval from the authors’ institutional ethics committee.
